# Parotitis and Gastric Ulcer

**Published:** 1904-12-17

**Authors:** 


### PAROTITIS AND GASTRIC ULCER.

A good deal of attention has been devoted in the last few years to the occurrence of inflammation of one or both parotid glands in the course of various illnesses originating in the intestinal tract, or during the period immediately following an abdominal operation. Though authorities differ in opinion with regard to the way in which the parotitis originates, probably the majority believe that the inflammation is due to infection spreading from the mouth up the parotid duct. In most of the conditions in which parotitis is liable to occur as a complication, the patient is either allowed to take no food at all by the mouth or else his diet is restricted to small quantities of liquid nourishment requiring no mastication and causing very little reflex secretion of saliva. Under these circumstances the oral cavity very readily becomes foul, and extension of organisms up the parotid duct is rendered less difficult than in conditions of health. Soltau Fenwick and H. Rhodes[1] give notes of three cases of parotitis occurring in patients who were under treatment for chronic gastric ulcer, having had attacks of hæmatemesis a few days previously. In each of these cases the first indication of disease was a complaint of pain in the face or the ear, with limitation of the movements of the jaw and the development of a tender swelling in the region of the parotid gland. A rise of temperature ensued, accompanied by increased frequency of the pulse. Indeed, the authors consider the pulse-rate to be a surer indication of the formation of pus than is the temperature. With regard to treatment they urge that as the inflammation is due to ascending infection of the parotid duct, it can be prevented by keeping the buccal cavity in a clean and moist state, and by allowing the patient, either to take food by the mouth, or to chew a piece of pellitory root or india-rubber in order to induce a secretion of saliva. In each of the three cases on which their paper is based the patient was being fed by the rectum to the exclusion of all nourishment by the mouth.

[1] Med. Press, Dec. 7.

